# Residents’ perceptions of an integrated longitudinal curriculum: a qualitative study

**Published:** 2015-12-11

**Authors:** Rebecca Lubitz, Joseph Lee, Loretta M. Hillier

**Affiliations:** 1 Andrew Street Family Health Centre, Kitchener, ON; 2 McMaster University, Hamilton, ON; 3 Centre for Family Medicine, Waterloo Region, ON; 4 Specialized Geriatric Services, St. Joseph’s Health Care London, and Aging, Rehabilitation & Geriatric Care Research Centre of the Lawson Health Research Institute, London, ON

**Keywords:** Medical education, competency-based curriculum, family medicine

## Abstract

**Background:**

The purpose of this study was to explore family medicine residents’ perceptions of a newly restructured integrated longitudinal curriculum.

**Method:**

A purposeful sample of 16 family medicine residents participated in focus group interviews conducted from a grounded theory perspective to identify the characteristics of this training model that contribute to and that challenge learning.

**Results:**

Eight key themes were identified: continuity of care, relevance to family medicine, autonomy, program-focused preparation, professional development as facilitated by role modeling, patient volume, clarity of expectations for learners, and logistics. Positive learning experiences were marked by high levels of autonomy, continuity, and relevance to family medicine. Less favorable learning experiences were characterized by limited opportunities for continuity of care, limited relevance to family medicine practice and unclear expectations for the resident’s role. Family physician-led learning experiences contributed to residents’ understanding of the full scope of family medicine practice, more so than specialist-led experiences. The logistics of implementing the integrated block were challenging and negatively impacted continuity and learning.

**Conclusions:**

This study suggests that an integrated longitudinalized family medicine block training model has the potential to support the principles of a longitudinal integrated competency-based curriculum to effectively prepare residents for family medicine practice.

## Introduction

Family medicine programs in Canada are implementing the Triple C curriculum, a competency-based curriculum marked by the key features of comprehensiveness, continuity, and centered in family medicine.^1^ This restructuring of traditional residency programs is supported by educational theory,^2^ expert opinion, and key leaders in family medicine.^1^,^3^ In 2009, the Kitchener-Waterloo site of the McMaster University Family Medicine program restructured its residency program consistent with the principles of a Triple C curriculum.^2^, ^4^ This site is situated in a predominantly urban area in Southern Ontario which incorporates the cities of Kitchener, Waterloo, Guelph, and Cambridge and the surrounding rural area. The area’s population is approximately 750,000, with four major secondary hospitals. This site began its residency program in 2005 consisting of a primarily block-based curriculum with two horizontal components operating throughout the two year duration of the program. Block, or rotational, models of training emphasize learning from specialists in sequential, discipline specific blocks of time in application to the family medicine content.^5^, ^6^

Curriculum restructuring in this program was driven by a desire to increase teaching capacity to support program growth, which increased from four first year residents in 2005 to 17 in 2013. A large number of family physician faculty were recruited thus limiting the number of specialist physicians required to teach. The curriculum change was driven by the perception that a family medicine-centred curriculum would enhance professionalism, socialize residents into the culture of family medicine, and support clinically relevant learning in medical and psychosocial domains.^3^ Focusing on teaching experiences that were rated on annual program evaluation surveys as highly relevant to family medicine residents, the new Triple C curriculum attempted to centre key content areas within the domain of family medicine using increased horizontal experiences within a novel integrated family medicine block and revising the block experiences that remained (Table 1). Within this integrated family medicine block, family medicine led experiences (newborn care, maternal/child health clinics, OB call, memory clinic, and long-term care) were included as horizontal experiences. These horizontal experiences were chosen because they are traditionally considered within the scope of family practice, and the family physician preceptors for those experiences were role-models for the practical integration of these areas into daily family practice. The specialist led experiences (developmental paediatrics, geriatric consults, internal medicine clinics) included in the integrated family medicine block were intended to provide additional depth to support the family medicine led experiences in the overarching domain (i.e. geriatrics; memory clinic with a family physician lead and supported with specialist consults) so that residents would have an appreciation for the boundaries of the family physician’s role and responsibilities in that domain as well as appropriate referral patterns. A detailed description of this curriculum is presented elsewhere.^7^

The internal medicine block was modified to a hospital medicine block primarily taught by family physicians specializing in hospital medicine. Components of speciality rotations (geriatrics, paediatrics, obstetrics & gynaecology, and internal medicine) were arranged horizontally into a two month integrated family medicine block, required in both Year 1 and Year 2 of the program in addition to the traditional core family medicine rotation. Time in traditional block rotations in those core areas was reduced, and that content area was re-introduced in a shared and longitudinal fashion between specialists and family medicine preceptors within integrated blocks of time. These integrated learning experiences provide opportunity for enhanced learner-patient relationships and patient-centred care,^8^ and facilitate competency specific to family medicine.^9^ Block experiences taught by specialists require the specialist physicians to ensure that the experience was orientated towards teaching residents that would become family physicians rather than physicians of that speciality.

There is a paucity of research on both longitudinal and family medicine-focused curriculums. Much of the research on longitudinal curriculums has been conducted at the clerkship level.^8^,^10^–^12^ There is some suggestion that in a longitudinal model with more continuity of care over time, learners are more motivated to learn and develop professionalism.^8^,^10^

Whether longitudinal curricula enhance continuity and learning remains debateable.^5^,^13^ There is little evidence to suggest that a particular way of educating residents is superior to another; repeated evaluations of residency and clerkship programs that have changed from a block-based curriculum to a longitudinal one have shown no difference in exam scores and tests of competence.^12^,^14^,^15^ A preliminary survey-based evaluation of the new integrated longitudinalized curriculum at the Kitchener-Waterloo site found that residents believed it effectively prepared them for family medicine practice;^7^ the factors that contributed to positive learning experiences in this training model were not clear.

This study explores family medicine residents’ perceptions of the newly restructured integrated family medicine curriculum within a distributed medical education program to identify the characteristics of this training model that contribute to perceived knowledge acquisition and professionalism.

## Methods

A qualitative study was undertaken using grounded theory to guide the analysis of residents’ perceptions of their learning experiences. This study was approved by the McMaster University Research Ethics Board.

### Participants

All residents enrolled in the 2010/2011 academic year (*n* = 26) were invited to participate in semi-structured focus group interviews. All of the residents were currently in the new integrated family medicine block; the first year of the second-year residents’ program was implemented in previous rotational model of training. The majority of interview participants had been exposed to the full complement of learning experiences available across various practice domains.

### Data collection

The focus group interviews were led by a third-year family medicine resident with research experience in qualitative methods. Interview questions were designed to elicit residents’ perceptions of how integrated block learning experiences supported their learning, their general impressions of the integrated experience, their perceptions of how their learning experiences within the integrated areas of practice such as allied health clinics, care of the elderly, women’s health, paediatrics, and internal medicine consult clinics supported their learning objectives and goals, future career choice, and overall professional development. Thematic saturation occurred after the third group interview as agreed upon by the two coders; data collection ended with the fourth focus group interview. The interviews were approximately 60 minutes in length and were audio-recorded and transcribed verbatim.

### Data analysis

Transcripts were analyzed independently by two researchers using a qualitative naturalistic inquiry approach^16^ to create an initial code book. This initial coding allowed for the identification of broad categories and emerging themes, which, with multiple readings of the transcripts, were adjusted to achieve greater clarity in the data.^17^ Participants’ responses to questions as related to each focused practice area/domain were first grouped together and notes were made of whether the focused area was experienced as positive or negative. A code book was then developed using grounded theory and standardized coding techniques to reflect the emergent themes that surfaced as residents’ voiced reasons for having positive or negative experiences in the various practice areas.^18^,^19^ These underlying reasons for positive or negative associations became the main themes in an expanded code book, which was then used to recode the transcripts in NVIVO 10.0 (QSR International). All transcript data was then coded on three levels: the practice domain in question, whether the comments were mainly positive or negative, and the underlying reason, or theme, if applicable. The coding stripes function in NVIVO was then used to cross reference these three distinct levels of codes. Particular attention was paid to looking at each focused area’s association with positive or negative experiences as well as the underlying reasons for that association. Final coding was compared to field notes created in the initial coding process to confirm emerging themes and new directions in the data.^20^

## Results

Sixteen residents (12 first-year, 4 second-year) participated in one of four group interviews, consisting of two to five residents per group. Eight key themes, or underlying reasons behind positive and negative associations, were identified. These themes were: continuity of care, relevance to family medicine, autonomy, program-focused preparation, professional development as facilitated by role modeling, patient volume, clarity of expectations for learners, and logistics. Quotes illustrating these themes are presented in Appendix 1.

### Continuity

Continuity in clinical and educational experiences was perceived as valuable to learning, particularly as it facilitated the development of a structured approach to care. Residents desired more clinical continuity than an eight-week block could provide, but were satisfied with opportunities to follow pregnant women and their babies over time in the maternal child health clinics, and with opportunities for continuity in the repeating long term care, geriatrics, and pediatrics psychiatry clinics. Residents were critical of learning experiences in which there was minimal continuity. As an example, in the geriatrics clinic, it was not a helpful learning experience to conduct a comprehensive assessment without being able to follow-up with the same patient. Conversely, it was not helpful to conduct follow-up visits with patients they had not previously assessed or seen, as occurred in some specialty clinics (e.g., paediatric psychiatry). Clinical discontinuity challenged some residents to understand their role in these practice areas. In terms of continuity of education, opportunities in weekly clinics to become familiar with faculty were viewed positively as rapport and learning expectations became well established. However, they noted that educational continuity sometimes jeopardized clinical continuity. Residents found that the repeating once weekly nature of newborn care over the entire two month block provided them with enough repetition to master skills and increase comfort level, but at the expense of clinical continuity, as there was no opportunity to follow babies to discharge over their typically short 48 hour stay.

### Relevance to family medicine

Areas in the integrated block that were most well received were those centred in family medicine, particularly the long term care experience, memory clinic, maternal and child health clinics, newborn care, anti-coagulation, optometry, and internal medicine consult clinics. All but the latter three were run by family medicine preceptors. These experiences were described as facilitating a structured approach to common family medicine problems. Specialty instructors in the latter three areas facilitated this type of learning by being aware and supportive of family medicine goals, targeting teaching experiences to residents’ learning needs, and being intentional in selecting opportunities most relevant to family practice. Depth of learning was influenced by relevance, as for example, with specialist led paediatric consult sessions, which were described as focusing on more complex cases. Residents perceived the depth of paediatric learning on the integrated block as more limited in comparison to their core paediatric rotation but that the learning was more relevant to family medicine.

### Autonomy

Residents valued learning opportunities that provided a high degree of autonomy, with supervision, as for example, in the anti-coagulation clinic where residents played an active role in reviewing and recommending warfarin dosing in collaboration with a pharmacist.

### Program-focused preparation

Residents valued clinics in which they received some introductory preparation. As an example, in the memory clinic they received a pre-clinic tutorial that provided specific content knowledge, assessment training, and direction regarding clinic structure and the roles and responsibilities of all team members. This pre-clinic preparation allowed residents to focus immediately on providing care instead of spending their time trying to ascertain how the clinic worked and what their role was. Residents described this preparation as assisting them to *“hit the ground running,”* function as managers early on, and maximize their learning in a short time span.

### Professional development as facilitated by role modeling

Learning experiences led by family physicians were highly valued for positive role modelling, facilitating beliefs that various practice areas could be integrated into their own future practice. These experiences provided residents with an opportunity to clarify scope of practice and procedural expectations within the context of family medicine versus specialty practice, and to elucidate lifestyle issues that influence practice choices. This was especially note-worthy in the family medicine-led obstetrics clinic, where residents felt deliveries with a family medicine preceptor were far superior for understanding the skill-level expectations of family doctors practicing obstetrics, deciding whether or not to practice obstetrics, and what that would entail. Specialist instructors who encouraged family medicine specific goals and practices were valued as supporting their professional development as family physicians. Learning situations not led by physicians, such as the allied health clinics, were often not considered positive as residents assumed an observer role in the absence of a physician-resident tutelage structure.

### Patient volume

Positive learning experiences were described as those with a good volume of cases from which to learn. Residents perceived that the patient volume in some allied health clinics was often too limited for this to be a high yield learning experience.

### Clarity of expectations for learners

Learning experiences were maximized when resident roles and responsibilities were clearly articulated. In the allied health clinics, residents reflected a mismatch between their skills and the ability of allied health practitioners to identify and articulate resident-appropriate roles. The exception to this was the anticoagulation clinic, where clear goals for residents’ involvement in monitoring anticoagulation therapy were identified as building their confidence in this practice area.

### Logistics

The logistics of the integrated block were described as challenging and likely detracted from educational continuity. Many residents found that having to go to different sites for clinics, sometimes several in one day and in a short time frame, made for a stressful experience and tied up cognitive resources in simple logistics (checking schedules, reorienting attention) and made preparation and consolidation of learning on any one topic problematic. For some residents, the changing nature of their schedules over the two month blocks, with new faces and environments to become familiar with, increased their anxiety and distracted them from learning.

## Discussion

In line with the rationale behind the Triple C Curriculum endorsed by the College of Family Physicians of Canada,^4^ the transition from a traditional block or rotational training model to an integrated family medicine block was intended to provide comprehensive exposure to core features of family medicine, provide more opportunities for continuity of care and education through expanded involvement in longitudinal family medicine clinics, and a centering of all these experiences in the family medicine context. Residents appreciated that the integrated curriculum exposed them to various patient care settings across the life span – from newborns in the hospital to geriatric patients in long term care centres – thus providing them the opportunity to learn how family physicians, with specialist support, provide comprehensive patient care. The results of this study have provided insights into the characteristics of the integrated, longitudinalized family medicine training model that have the potential to facilitate optimal learning and those that pose a barrier to residents’ knowledge and skill acquisition. Residents reported that in this training model continuity of care, relevance to family medicine, autonomy, program-focused preparation, professional development as facilitated by role modeling, patient volumes, clarity of expectations for learners, and scheduling logistics, impacted their learning. Some of these themes are not necessarily unique to integrated longitudinalized training models, such as autonomy, program-focused preparation, patient volumes, clear expectations and logistics. However, continuity of care, relevance to family medicine, and professional development as facilitated by role model are more directly impacted by this longitudinalized curriculum.

Curricula centred in family medicine, in which learners are taught by family physicians within primary care settings, have been described as most optimal for promoting the achievement of core competencies for family medicine practice^4^ and to socialize residents to the professional identity and role of family physicians.^21^ In this study, practice areas in the two month block that residents valued most were those that provided learning experiences that were relevant to family medicine, offered clinic-specific preparation to help residents maximize the learning experience, promoted autonomy, had an adequate volume of patients, and exposed residents to positive role modelling. Curricula centred in family medicine, in which learners are taught by family physicians within primary care settings, have been described as most optimal for promoting the achievement of core competencies for family medicine practice^4^ and socializing residents to the professional identity and role of family physicians.^21^ Specialist instructors facilitated meaningful learning when learning opportunities were focused on those most relevant to family practice. In doing so, residents gained a more realistic understanding of the potential scope of practice in the specified area and when it is appropriate to refer to specialists.

From a health system perspective, this ensures that specialist resources are utilized appropriately and not for issues that can and should be managed optimally in primary care. This constellation of attributes highlights the importance of learning experiences centred in family medicine, even when led by specialists. Similarly, in learning situations led by allied health professionals, residents reported that they learned most when their role as a resident physician was clearly stated and when they were actively engaged (“practicing” as in the anti-coagulation clinics), as opposed to simply observing the practice of others as was the case in other allied health clinics.

Physician training has been criticized in the past for the lack of continuity and connection between different learning experiences.^22^ The lack of sustained relationships with patients and educators has prompted the development of a number of different training models to better promote continuity.^10^ Integrated horizontal training models have been identified as an opportunity to develop core competencies by linking learning experiences between and across various practice domains.^10^ In this study, opportunities for continuity impacted residents’ perceptions of their learning experiences. Most notably, they desired more clinical continuity than two month blocks could provide. Practice areas in which there was limited continuity were perceived less favorably than those with greater continuity. Limited continuity existed in clinics such as geriatrics and pediatric psychiatry where residents conducted comprehensive assessments but never had the opportunity to follow patients to evaluate the effectiveness of their treatment recommendations. In contrast, the maternal and child clinics provided greater continuity as residents followed mothers for prenatal and postpartum care and provide care to their newborns. These opportunities for continuity of care are critical for promoting the full range of clinical skills needed to provide patient-centred health care.^10^ Clinical discontinuity has been described as inefficient and frustrating for learners.^23^ Consistent with this, residents in this study reported that limited continuity of care made it difficult for them to understand their role within the practice domain. In terms of continuity of education, residents valued once-weekly clinics that spanned the two month integrated block due to improved opportunity to establish rapport with instructors over a longer period of time and to become familiar with the learning environment’s routines and expectations. However, this educational continuity was noted, in some practice areas (e.g., inpatient newborn care), to minimize opportunities for clinical continuity.

The logistics of managing competing priorities as related to outpatient and inpatient responsibilities compromises continuity of care and education.^10^ It has been noted that integrated horizontal training models require greater logistical management than traditional rotational models. The logistical complexity associated with managing faculty and learners in various clinical sites and those associated financial implications are a key challenge with this training model. In this study, the logistics of implementing the integrated block were viewed as negatively impacting educational continuity and learning. Multiple clinics in a week, with multiple instructors, were identified as a barrier to learning for some residents as they perceived that a significant portion of their time was spent traveling to various clinic sites and figuring out expectations and logistics, which distracted them from the real work of learning content. These logistical challenges could potentially be minimized by developing consistency in clinic scheduling: schedule focused areas on the same day and time weekly, and replace once weekly clinics with daily (half day) clinic opportunities, which would enhance clinical continuity in some areas (e.g. newborn care) but may be less appropriate for continuity in areas such as paediatric consultation and psychiatry where patient follow-up occurs over a greater length of time.

### Limitations

Although a majority of the residents enrolled in the Kitchener-Waterloo site volunteered to participate in this study, the potential for selection bias exists so that the results may not be representative of the entire resident population. Although the sample size is small and the majority of participants were first year residents who did not have experience with the previous rotational training model, the residents in this study nonetheless provided valuable information about their experiences with the integrated block model. Although disclosure within the groups may have been impacted by the leader being known to some of the participants, this was likely minimal given the range of positive and negative experiences disclosed and the consistency in themes identified across all four group interviews. As only four residents had experience with previous rotational training model, direct comparisons with the integrated model were not possible.

### Conclusions

The results of this study suggest that an integrated, longitudinalized family medicine block has the potential to effectively support the principles of a Triple C curriculum as experienced by residents. Careful attention to logistics should be a key focus for program directors, as the implementation of this curriculum impacts the perceived quality of learning experiences, particularly related to continuity and comprehensiveness of learning. The results of this study can inform program improvements to optimize learning and professional development in family medicine. Further study is required to provide empirical evidence of the role of this curriculum on specific outcomes such as knowledge and skill acquisition and career and practice choices.

## Figures and Tables

**Table 1:** Former ‘block’ curriculum and the new integrated family medicine block

Former curriculum Behavioural Science (one half day a week) Resident Rounds (one hour per week)						
Year 1	Family Medicine (4 months)	Hospital Medicine (2 months)	Pediatrics (2 months)		Ob/Gyn (2 months)	Emergency (2 months)
Year 2	Family Medicine (4 months)	Rural Family Medicine (2 months)	Geriatrics (1 month)		Internal Medicine/Surgery Selectives (3 months)	Electives (2 months)

**New integrated family medicine curriculum** Behavioural Science (one half day a week) Resident Rounds (one hour per week)						

Year 1	Family Medicine (4 months)	Integrated Family Medicine (2 months)*	Hospital Medicine (2 months)	Pediatrics (1 months)	Ob/Gyn (1 month)	Emergency (2 months)
Year 2	Family Medicine (4 months)	Integrated Family Medicine (2 months)**	Rural Family Medicine (2 months)		Electives (2 months)	Integrated Family Medicine (2 months)**

* Pediatrics (newborn care, paediatric consults, developmental paediatrics, paediatric psychiatry); OB/GYN (OB call with family physician call group, maternal/child health clinics and gynecological procedures clinic); and Allied Health Clinics within the family medicine centre (dietitian, anticoagulation clinic, optometry sessional, diabetes, and congestive heart failure clinics, smoking cessation, asthma and chronic obstructive pulmonary disease clinics, memory clinic) were longitudinalized in the integrated block in Year 1. Note: newborn care, OB on-call, and maternal/child health clinics are led by family physicians.

** Geriatrics (long-term care, outpatient and inpatient consults), internal medicine consult clinics, Allied Health Clinics within the family medicine centre (congestive heart failure and diabetes clinics, smoking cessation, asthma and chronic obstructive pulmonary disease clinics, memory clinic) were longitudinalized in the integrated block in Year 2. Note: long-term care and the memory clinic are led by family physicians.

## Appendix 1

**Table:** Focus group participants’ quotes related to themes, illustrating positive and challenging learning experiences associated with the integrated curriculum

Theme	Positive learning experiences	Challenges to learning
Continuity	*“Newborn care was very good, especially because it was continuous through the two months, so you saw it enough that you actually became comfortable with it, and developed a routine and an approach that you can take away.”* [Newborn Care;FG4ID3] *“...Just having the repetition of normalcy, will allow us as doctors later on to really be able to pick up when something just does not seem right, like you have heard enough fetal heart rates now that you should be able to figure out when something is not right here, or mom is not measuring properly.”* [Maternal Child Health Clinics; FG4ID2] *“ I had combination* [visits of] *both well baby and the post-partum check, and it is ... nice for the family but it is also nice for you as the family physician looking after them, and you start to feel more comfortable with both.”* [Maternal Child Health Clinics; FG3ID1] Educational continuity: “[Internal medicine consult clinic] *was extremely useful, very well organized ... You always had a patient set up for you, and then* [the staff physician] *would follow up ... there was always enough time to do a discussion and then if there was extra time, he* [staff physician] *was really good about teaching you. So if you brought a topic, he would, on the fly just go through things with you.”* [Internal Medicine; FG2, ID1]	*“....there was some follow up – but I didn’t know anything about the patient ... it was more like, how you doing, okay here’s your prescription. I was just watching ... I don’t know the whole story. So I didn’t find that as useful, as the new consults were useful. A lot of the time, when she would give me the chart, it was like, “Oh, review while I go grab the patient,” And you literally have 2 minutes, until they walk the patient from the waiting area. – to figure out why they are there and what you are following up on.”* [Paediatric Psychiatry; FG2ID2] *“The in patients sometimes wouldn’t necessarily be there next week. You never know what happened to them, so it’s not very helpful.”* [Hospital Medicine; FG1ID4]
Relevance to family medicine	*“He* [the optometry instructor] *guided the session towards the needs of myself .... instead of focusing on all of their fancy devices, he actually used an ophthalmoscope and went through things with that approach as opposed to what they normally do, and even just talking about diseases in the context of family medicine and when you might refer.”* [Allied Health Clinics; FG1ID5] *“They* [Specialists] *were good at inspiring confidence at doing things family doctors can and should be able to do.”* [Women’s Health; FG4ID1] “[The integrated block] *was a lot more focused towards family medicine, because doing an inpatient rotation on a paeds wards ... you may feel like you learned more about paediatrics coming out of it, but it is not going to be applicable to your everyday practice.”* [Paediatrics; FG3ID2]	*“It* [family medicine focused obstetrics] *helps you consider doing obstetrics in your practice whereas if you were to do just obstetrical clinics, the volume and complexity of cases might discourage most people from considering it.”* [Obstetrics; FG4ID3] “*I have friends (in other programs) who are in the NICU for one month, and they* [found it] *completely useless, I am never going to need this [advanced NICU training] at all, what is the point, so I feel really lucky that we have what we have.”* [Paediatrics; FG4ID2]
Autonomy	*“There’s lots of support there but it’s really the residents there that run the clinic and then you review. If there’s any procedures, one of the residents there is doing it.”* [Obstetrics; FG3ID1] *“Sometimes you are there by yourself and making the decisions by yourself, which is completely fine. I found you have a lot of autonomy. You get to work with the nurses and they get to know you over time and it’s really enjoyable.”* [Long-Term Care; FG3ID3]	*“You know, for a second year resident to sit and watch the dietitian for four mornings doing her phone business is not as useful as trying to do the dietary counseling. I think doing one observation and then trying to do it yourself…. So being an observer was really most frustrating about that experience.”* [Allied Health Clinics; FG1ID2] *A little less useful is the Chronic Disease Management. A lot of the time is spent observing …. I felt that it wasn’t the densest learning sitting, kind of watching.”* [Allied Health Clinics; FG3ID2]
Program focused preparation	*“You are not going in to try to learn how things work and what types of things you are looking for, you already have that background information and you’re looking more at management and how you’re going to treat this person and help their family ... It was useful to have that primer so you could just go in and do the most important stuff.”* [Memory Clinic; FG1ID3] *“The first week was really good ... because she went over all the major algorithms like jaundice and blood sugar, so that made it more comfortable ...”* [Newborn Care; FG4ID3]	*“It’s difficult as a resident* [to] *take over, when you don’t know what you are doing or what the purpose of the clinic is, and then you never go back to actually go in and take that role as a take-over.”* [Allied Health Clinic; FG2ID3] *“I feel like that* [pre-clinic preparation] *would be helpful ... if you knew what paeds consults you were going to see in the other sessionals ... you could read about it and develop your own approach and then see how the paediatrician would do it differently – rather than panic and try to say, I have no clue what I am doing, I’m going to just try my best and then sort of feel overwhelmed.”* [Paediatric Sessional Consult Clinic; FG4ID1]
Professional development as facilitated by role modeling	*“I said: I can see myself doing this one day because the family doctor, he comes in and does this.”* [Long-Term Care; FG1ID4] *“So it is important to see. It* [family physicians in specialty clinics] *can help make a resident make a decision: Yes or no, Is this something that they would like to incorporate into their practice and how much?”* [Obstetrics/Gynaecology; FG3ID1] *“Actually, yes, I want to do OB know. Meeting the call group in town and working with them, and ... do*[ing] *a lot of deliveries with them -- in* [one] *month I got 7* [deliveries] *- I think it was exposure to the way that the physicians here do it – ... how they do it, and how it works for their life, and just seeing people actually do it [that helped me make my choice to do obstetrics].”* [Obstetrics/Gynecology; FG4ID4] *“I think it’s good to see [family physicians in specialty clinics]. As a family doctor you can kind of structure your practice in that way so it’s good exposure for us.”* [Memory Clinic; FG3ID2]	*“It seems weird to me that in OB clinic, when the baby is sitting there in the basket for the post-partum check and the OB does not even look at it. It made a lot more sense, and it was so much easier on the moms who came in, when they had their well-baby check and their post-natal check at the same time, so a little more work on us, but both need to be done.”* [Obstetrics Clinics vs Maternal Child Health Clinics; FG4ID1] *“Geriatrics, useful in some ways but not for that particular goal* [mimicking family physician role]*. Because as a family doctor you are never going to be the geriatric consultant.”* [Geriatrics; FG1ID5]
Patient volume	*“I really liked the anti-coagulation clinic. It was high volume and very concentrated, and I found I really had no clue going into it how to manage INRs* [International normalized ratio index of blood coagulability] *and warfarin dosing, and coming out of it I was a lot more confident ... that was something I had not had before in teaching and it was really useful.”* [Allied Health Clinics - Anti-Coagulation Clinic; FG4ID2] *“I got a lot of volume of prenatal patients early on in the pregnancy... it was useful to get that extra volume, to see some of the more unusual things from the perspective of primary care physicians with all the other information, knowing all the patients for quite some time.”* [Maternal Child Health Clinics; FG1ID2]	“*But the fact that, they sometimes didn’t have a reasonable number of patients to really deal with or get anything out of it. There were a couple of times where I saw one patient in the whole afternoon. Which could have been a better use of my time in doing something else.”* [Allied Health Clinics; FG1ID3] *“On that block she [Physician] gave you every full day with 3 patients and that’s how it was booked. Towards the end, I felt like I would get more out of my family practice clinic, seeing 12 patients in a half day than I would with 3 patients for a full day. It just felt like the volume wasn’t there.”* [Geriatrics; FG1ID4]
Clarity of expectations for learners	“*Good staff … are able to have an open dialogue with the resident with what the expectations are.”* [Obstetrics/Gynaecology; FG3ID3] *“I found that with [Physician], we reviewed what cases were coming in to the clinic the following week, to preview what was coming in, whether it was query autism or something really weird that I had never heard of before. So I would go home and read it. And then, that was a great way to prepare, because I’d come in, and know what kinds of physical signs we were looking for and which was the important part of that day. So that was an example that worked really well, for having expectations and some lead guidance.”* [Developmental Clinic; FG2ID3]	*“It took a lot to figure out my role. And then it wasn’t fairly simple, it wasn’t until 4 or 5 weeks that I actually started booking follow-ups. It took it awhile to make it a good experience.”* [Paediatric Psychiatry; FG2ID1] *“I was there as an observer only, and I think that it was more appropriate for a medical student as opposed to a resident. I think focused reading and fixed objectives would be quite helpful for both the resident and the staff, so that they need to know, ‘Ok, this is what the resident needs to learn, this is what they need to do in order to practice - to provide that care.’”* [Allied Health; FG1ID7] *“It would be really nice to have orientation material, with a brief handout of what you can expect on your first day: ‘Here is a short amount of reading to prepare yourself.’ Instead of: ‘Wow, I have newborn care, I should probably read up everything that could happen with a newborn.’ because we are just not sure what to expect and what the expectations on us are, the first day.”* [Paediatric Psychiatry; FG2ID2]
Logistics of the integrated block (Challenge to learning)	*“Learning wise, I think it takes a bit of a toll as well, because when you do 8 different clinics in a week, then the next week you almost have to remember where to walk into the clinic, how to get there, let alone what you’re actually doing in the clinic and what your goals are.”* [FG2ID1] *“I found it very difficult to try to prepare. You show up to gyne clinic on Monday and you gotta be “gyne,” and then the next day I have to read about newborn care and I want to review hypoglycemia and bilirubin. Then the next day I’m at* [developmental clinic]*, I have to read* [about development issues].” [FG2ID3]	

## References

[1] Saucier D, Shaw E, Kerr J (2012). Competency-based curriculum for family medicine. Can Fam Phys.

[2] Weston WW (2012). Underlying educational principles of Triple C. Can Fam Phys.

[3] Shaw E, Walsh AE, Saucier D (2012). The last *C*: centred in family medicine. Can Fam Phys.

[4] Tannenbaum D, Kerr J, Konkin J (2011). Triple C competency-based curriculum. Report of the Working Group on Postgraduate Curriculum Review - Part 1.

[5] Weiss BD (2001). Longitudinal residency training in family medicine: not ready for prime time. Fam Med.

[6] Reust CE (2001). Longitudinal residency training: a survey of family practice residency programs. Fam Med.

[7] Lee J, McMillan C, Hillier LM, O’Brien G (2013). Preparing residents for family practice: the role of an integrated “Triple C” curriculum. Can Med Educ J.

[8] Ogur B, Hirsh D (2009). Learning through longitudinal patient care-narratives from the Harvard medical school - Cambridge integrated clerkship. Acad Med.

[9] Cox M, Irby D (2007). “Continuity” as an organizing principle of clinical education reform. N Eng J Med.

[10] Hirsh DA, Ogur B, Thibault GE, Cox M (2007). Continuity as an organizing principle for clinical education reform. N Eng J Med.

[11] Norris TE, Schaad DC, DeWitt D, Ogur B, Hunt D (2009). Longitudinal integrated clerkships for medical students: an innovation adopted by medical schools in Australia, Canada, South Africa, and the United States. Acad Med.

[12] Ogrinc G, Mutha S, Irby D (2002). Evidence for longitudinal ambulatory care rotation: a review of the literature. Acad Med.

[13] Steinweg KK, Cummings DM, Kelly SK (2001). Are some subjects better taught in block rotation? A geriatric experience. Fam Med.

[14] Reust CE, Stehney M (2001). Models of innovation: longitudinal curriculum in family practice education. Society of Teachers of Family Medicine Monograph.

[15] Worley P, Silagy C, Prideaux D, Newble D, Jones A (2000). The parallel rural community curriculum. Med Ed.

[16] Lincoln YS, Guba EG (1985). Naturalistic inquiry.

[17] Braun V, Clarke V (2006). Using thematic analysis in psychology. Qual Res.

[18] Walker D, Myrick F (2006). Grounded theory: an exploration of process and procedure. Qual Health Res.

[19] Patton MQ (2002). Qualitative evaluation and research.

[20] Berg BL (2001). Qualitative research methods for the social sciences.

[21] Beaulieu MD, Rioiux M, Rocher G, Samson L, Boucher L (2008). Family practice: professional identity in transition. A case study of family medicine in Canada. Soc Sci Med.

[22] Glick TH, Moore GT (2001). Time to learn: the outlook for renewal of patient-centred education in the digital age. Med Ed.

[23] Irby D (2007). Educational continuity in clinical clerkships. N Eng J Med.

